# Epithelial to mesenchymal transition induces stem cell like phenotype in renal cell carcinoma cells

**DOI:** 10.1186/s12935-018-0555-6

**Published:** 2018-04-11

**Authors:** Mamta Singla, Ajay Kumar, Amanjit Bal, Subhendu Sarkar, Shalmoli Bhattacharyya

**Affiliations:** 1 Department of Biophysics, Postgraduate Institute of Medical Education & Research, Chandigarh, India; 2 Department of Histopathology, Postgraduate Institute of Medical Education & Research, Chandigarh, India; 3 Department of Physics, Indian Institute of Technology, Roopnagar, Roopnagar, India

**Keywords:** Renal cell carcinoma, Cyclosporine A, Transforming growth factor-beta, Epithelial to mesenchymal transition, Chemoresistant gene, Cancer stem cells

## Abstract

**Background:**

Metastatic dissemination of solid tumors is often initiated by reactivation of an embryonic development program, epithelial-to-mesenchymal-transition (EMT). EMT has been associated with acquiring invasiveness and resistance to conventional therapies. However, the precise role of EMT during renal cell carcinoma is still debatable and is under investigation. In this context, our study is designed to evaluate the role of cyclosporine (CsA) and transforming growth factor-β (TGFβ) administration in inducing EMT-like state in renal carcinoma cells. We also studied the associated phenotypic changes which may lead to tumor metastasis.

**Methods:**

The morphological changes in renal cell carcinoma cells (A498) treated with TGF-β/CsA were observed by microscopy. Atomic force microscope was used to evaluate the changes in elasticity of cells treated with TGF-β/CsA. The expression of mesenchymal and chemoresistance genes were checked by RT-PCR. Assays for migration, invasion, sphere formation ability and expression of cancer stem cell-like phenotypes were done to evaluate the metastatic potential of these cells. Lineage specific differentiations were also done to determine the acquisition of stem-cell like phenotype.

**Results:**

Our results showed that treatment with TGF-β/CsA led to loss of epithelial characteristics and gain of mesenchymal phenotype in vitro. Changes in shape and elastic properties of the cancer cells favoured metastatic progression, increased tumorisphere formation and invasiveness post treatment. We also observed higher expression of chemoresistance and stemness markers in EMT-induced cells. These cells also differentiated to various lineages like osteoblasts, adipocytes, neural and hepatic cells when induced with the respective differentiation media.

**Conclusion:**

We concluded that TGF-β/CsA treatment led to acquisition of EMT-like cancer stem cells phenotype that enhanced local invasion and dissemination of renal carcinoma cells. This subpopulation of cells with EMT-like phenotype a can provide a better perception of the metastatic process. This can provide an in vitro system for testing pharmaceuticals for modulating EMT as a viable strategy within the therapeutic armamentarium for RCC patients. The results of our findings also suggest that CsA directly induced EMT like changes in epithelial cell which may be responsible for the potential risk of malignancy in transplant patients.

**Electronic supplementary material:**

The online version of this article (10.1186/s12935-018-0555-6) contains supplementary material, which is available to authorized users.

## Background

Primary neoplasia is treatable, but metastasis pose a major threat to cancer patients. It is very important to understand the mechanism of metastases, especially to cure advanced cancers. Growing evidences suggest that the process that aids in the progression toward invasive and metastatic cancer is associated with the reactivation of an embryonic development program, epithelial-to-mesenchymal-transition (EMT) [1]. It is a complex process that regulates changes in cell morphology and mechanical properties of the cells. Modulation of elastic properties of cells during transition allows the cells to detach from their neighbours and the underlying basement membrane to facilitate their easy migration and invasion during metastasis [2]. During the process of tumor progression, a small fraction of cancer cells acquires self-renewal potential like stem cells and are referred as cancer stem cell (CSC). A reciprocal relationship between CSC and EMT might have a role in tumor progression. EMT is critical for acquisition of invasive behaviour and CSC properties, therefore effective therapies can be developed against metastatic cells via interference with the CSC/EMT differentiation program.

The association of carcinogenesis with organ transplantation is found to be complicated. CsA, an immunosuppressive agent is used during the organ transplant has been linked for cancer progression by cell autonomous mechanism [3]. There is increasing evidence that immunosuppressive agents like CsA may have unique oncogenic profiles [4]. A recent case study has shown that cyclosporine treatment to a psoriasis patient, led to multiple metastases [5]. CsA has also been shown to induce phenotypic changes in keratinocytes that can increase cell invasiveness and unregulated tumor growth [6]. CsA has also been associated with the promotion and stimulation of cellular proliferation in normal human fibroblasts [7]. However, the precise mechanism underlying the tumorigenic effect of CsA remains largely obscure though CsA is known to increase the level of TGF-β which is strongly involved in the regulation of EMT in cancer [8]. Higher production of TGF-β is positively associated with tumor aggressiveness and poor prognosis [9, 10]. However, there is no direct evidence that CsA may induce malignancy or cause increase in invasiveness or CSC-like phenotype. We designed our study on A-498 cells which is considered a “classical” clear cell renal carcinoma line with VHL mutation belonging to the NCI-60 panel. Clear cell carcinoma is the most prevalent subtype of RCC and is therefore widely used in cancer research. In this study, we observed that CsA could cause EMT in A498 cells leading to morphological change, increased invasiveness and stem cell-like phenotype. Thus, it is important to consider the possibility of malignant transformation during treatment planning in patients. The results of our study indicate that EMT plays a role in metastatic progression, drug resistance during RCC and targeting EMT may represent an effective strategy to combat the disease.

## Materials and methods

### Cell culture

In-vitro studies were done with A498 cell line obtained from NCCS, Pune, India and grown in the DMEM-high glucose (Dulbecco’s modified eagle’s medium) containing 10% FBS (Fetal Bovine Serum) and antibiotics (pen-strep, 1U/ml) at 37 °C at 5% CO_2_ in humidified incubator. Cyclosporine-A was obtained from Sigma Aldrich (USA).

### In vitro induction of EMT

5 × 10^4^ cells were seeded per well of 12-well plates and allowed to grow overnight. Next day, these cells were incubated with TGF-β (5 ng/ml) or CsA (10 μM) separately for another 48 h in the DMEM containing 10% FBS.

### Light microscopy

The morphological changes due to EMT induction were confirmed by observing under bright field microscope (Nikon).

### Atomic force microscopy

A multimode 8 atomic force microscopy (AFM) system (Bruker AXS) was used to image the control and treated cells and perform mechanical measurements on them. A large scanner having a maximum x–y scan range of 125 × 125 µm and a z-limit of 5 µm was employed to scan the surface. Measurements were done in tapping mode using a cantilever tip (SCANASYST-AIR) having a force constant of 0.4 N/m, prior to measurements, the cantilever was calibrated using the thermal vibration method. DMT modulus was calculated by using the load force and adhesion data. The reduced Young’s modulus (E) in this model was obtained by fitting the retract curve according to the equation $F_{tip} = \frac{4}{3}E\sqrt {Rd^{3} } + F_{adh}$. Here, F_tip_ is the force on the tip, F_adh_ is the adhesive force, R is the tip radius and d are the tip-sample separation.

### Staining with acridine orange (AO)

A498 cells (4 × 10^3^/well) were treated with CsA for 48 h. After treatment, live cells were washed with PBS and stained with AO (1 µg/ml) for 15 min. Cells were again washed in PBS and visualized using Fluorescent microscope (Olympus 1X51) under excitation and emission wavelengths of 488/532 nm.

### Immunocytochemistry

Cells were grown on cover slips, fixed with 4% paraformaldehyde for 15 min and permeabilized with 0.2% Triton X-100/PBS. The cells were then incubated at 37 °C with primary antibodies for cytokeratin (Dako, 1:200), vimentin (Dako, 1:100), E-cadherin (Dako, 1:100) for 1 h followed by horse radish peroxidase-conjugated secondary antibodies (Dako Envision) for 45 min at room temperature. Color was developed by adding HRP substrate, 3′3-diaminobenzidine and cells were counterstained with hematoxylin. The cells were observed under the light microscope (Leica DM 2500).

For immunofluorescence study, the cells were incubated with the human EMT 3-color immunocytochemistry kit (SC026) as per manufacturer’s instructions and observed under confocal microscope (Olympus). Images were acquired using FV1000 software at-10× and 20× magnification.

### Real time polymerase chain reaction (PCR)

Total RNA was isolated from the cells using Trizol reagent (Invitrogen) and reverse-transcribed using cDNA synthesis kit (Bio-Rad) per manufacturer’s protocol. Real-time PCR analysis was performed on a Roche real-time PCR system using the Power SYBR Green PCR Master Mix (Roche, Foster City, CA). The expression value of each gene was normalized against the amount of β-actin and calculated by the ΔΔC_t_ method. Details of the primer sequence are given in Table 1.

**Table 1:** The list of primers used for Real time PCR studies

S. no	Gene name	Sequence
1	β-Actin	F 5′CAAGAGATGGCCACGGCTGCT3′ R 5′TCCTTCTGCATCCTGTCGGCA3′
2	Vimentin	F 5′TCTACGAGGAGGAGATGCGG3′ R 5′GGTCAAGACGTGCCAGAGAC3′
3	Snail	F 5′GAAAGGCCTTCAACTGCAAA3′ R 5′TGACATCTGAGTGGGTCTGG3′
4	Slug	F5′ATTCGGACCCACACATTACCTTG3′ R5′TGGAGAAGGTTTTGGAGCAGTTT3′
5	Twist	F5′TGAGCAAGATTCAGACCCTCA3′ R 5′ATCCTCCAGACCGAGAAGG3′
6	ABCG2	F5′-CTGAGATCCTGAGCCTTTGG-3′ R5′-TGCCCATCACAACATCATCT-3′
7	MDR2	F5′-GCCTGGCAGCTGGAAGACAAATAC-3′ R5′-ATGGCCAAAATCACAAGGGTTAGC-3′

### Migration and transwell invasion assay

The cells were cultured up to 80–90% confluency and scratched with a pipette tip to create a uniform wound. Migratory ability of the cells was evaluated from the time taken in filling the open space created by scratch under inverted microscope. The wound area closure was calculated using the T-Scratch software developed by the Koumoutsakos group, Zurich [11].

For invasion assay, Matrigel™ (BD Biosciences, 1 mg/ml) was added to 24 well cell culture inserts (8 µm). Cells were seeded on the upper chamber of the inserts in serum free media. Media containing 10% FBS was added in the lower chamber and incubated for 48 h at 37 °C in a humidified 5% CO_2_ incubator. The migratory cells present on the lower surface were stained with 0.1% crystal violet and photographed.

### Colony formation assay

Single cell suspensions (1000 cells/well) were seeded on 6 well tissue culture plate in DMEM containing 10% FBS without any additional coating [12]. After 14 days of culture, the colonies were washed with phosphate buffer saline (PBS), fixed in methanol, and stained with 0.1% crystal violet. The stained colonies were observed under the microscope and photographed.

### Tumorisphere assay

Cells treated with and without cyclosporine (CsA) were cultured using DMEM-Ham’s F12 (1:1; Sigma) containing bFGF (basic fibroblast growth factor; 10 ng/ml), B27 (0.5%), EGF and 0.4% bovine serum albumin (BSA). These cells could grow for 7 days in low adherence plates (six well, Corning). Photography was done after 7 days to observe the tumorisphere formation ability of cells in presence or absence of CsA.

### Differentiation assays

The control and CsA treated cells were induced for differentiation to osteogenic, adipogenic and neural lineage using suitable differentiation media.

For osteogenic differentiation, cells were incubated in alpha-minimal essential medium (α-MEM) complete medium (CM), supplemented with 0.01 mM dexamethasone disodium phosphate, 1.8 mM monopotassium phosphate (KH_2_PO_4_) and 5 mM β-glycerophosphate. Media was changed every 3rd day. At the end of 21st day, the cells were stained with 1% Alizarin Red S (AR-S) for osteogenic differentiation.

For adipogenic differentiation, cells were exposed to adipogenic media made of αMEM containing isobutyl-methylxanthine (IBMX, 0.5 mM), dexamethasone (1 μM), insulin (10 μM), and indomethacin (200 μM). At the end of 21st day, the cells were subjected to oil red O staining for the presence of intracellular lipid droplets.

For neural differentiation, cells were incubated in neurobasal medium (Life technologies) pen/strep (1%), supplemented with B27, 20 ng/ml bFGF and EGF, N2 and G5 supplement. After 21st day, cells were fixed and incubated with anti-human neurofilament antibody (NFM, 1:50) (Sigma Aldrich), followed by FITC labelled secondary antibody (Sigma) and observed under fluorescence microscope (Nikon).

For hepatic differentiation, cells were incubated in αMEM containing ITS + (Invitrogen, 50 mg/ml) premix, EGF (epidermal growth factor; Invitrogen, 2 ng/ml), dexamethasone (0.5 μM) and HGF (hepatocyte growth factor, Sigma, 20 ng/ml) during induction phase (14 days). The maturation medium contained supplements like Oncostatin M (Sigma, 20 ng/ml), ITS and dexamethasone. Analysis of hepatocytes was done by low density lipoprotein assay kit (LDL assay kit-Abcam) as per instructions provided with kit. Fluorescent markers used in the kit include DyLight™ 550 and LDL receptor antibody-for LDL uptake and receptor distribution in the cells respectively.

### Statistical analysis

All experiments were performed at least in triplicates and were repeated three times. Data was reported as mean ± SD. All statistical analyses were performed using one-way ANOVA and paired t test.

## Results

### Induction of EMT in vitro

**Fig. 1:**
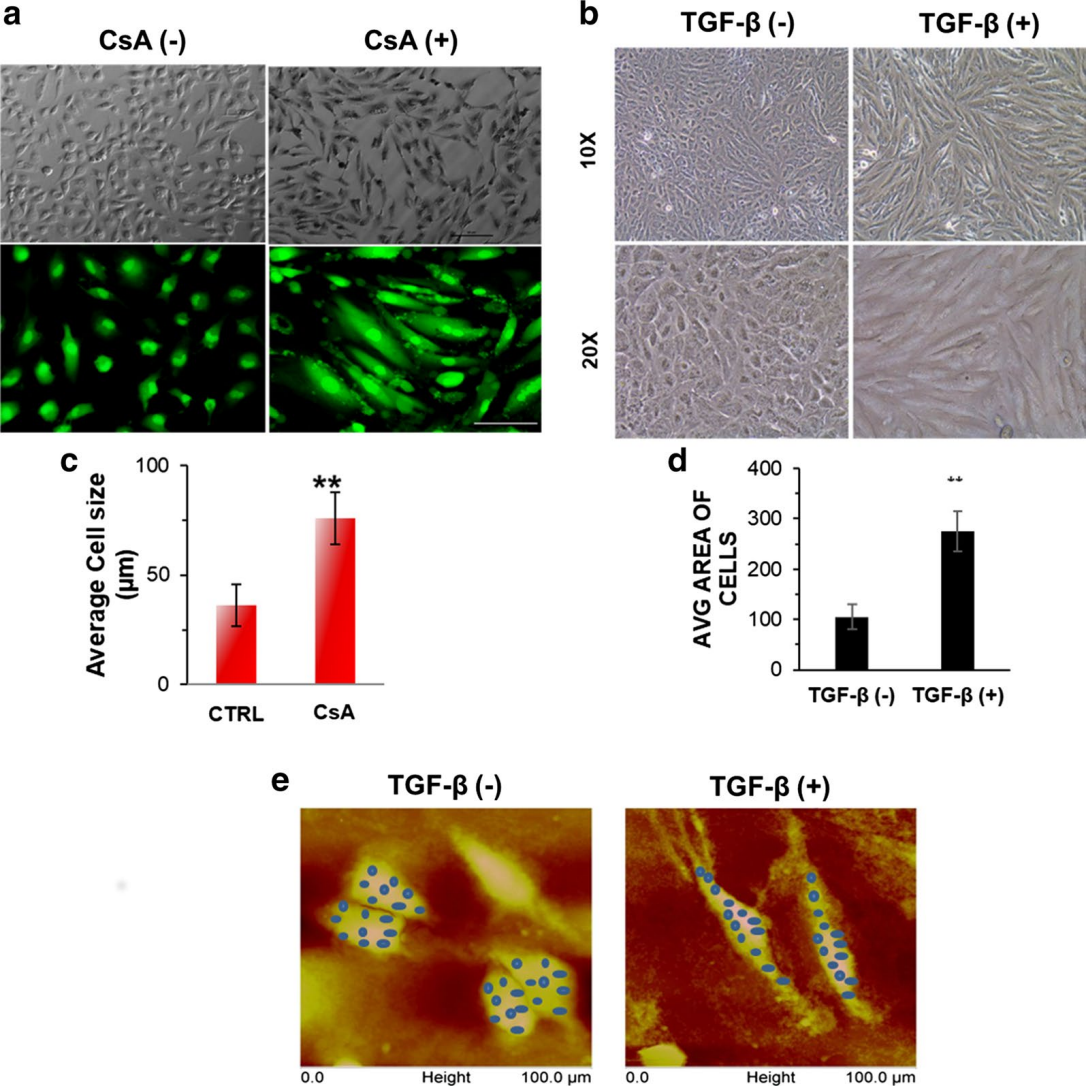
Morphological change in A498 cells. **a** Light microscopy images of A498 cells treated with CsA (10 µM) for 48 h. Magnification ×10. Scale bar—20 µm. Upper panel—bright field images of A498 cell morphology with or without CsA treatment. Lower panel—CsA treated A498 cells, stained with acridine orange and visualized under fluorescent microscope in a blue filter. Magnification—×10, ×20. **b** Change in cell shape with TGF-β treatment: A498 cells were treated with or without TGF-β (5 ng/ml) for 48 h. Bright field images showing change in morphology after treatment and cells acquire spindle shaped morphology. Magnification: ×10 and ×20. **c** Graphical representation of average cell size in control and CsA treated A498 cells. Statistical comparison represents average cell size in minimum 10 fields. Data is represented as mean ± SD. P value *< 0.05, **< 0. **d** Bar diagram showing comparison between average area of the cells with and without TGF-β treatment. Area of at least 20 cells were measured by using ImageJ software. Data is represented as mean ± SD. P value ***< 0.0001. **e** Atomic force microscopy images of A498 cells treated with or without TGF-β (5 ng/ml) for 48 h. Young modulus was calculated at different points (blue dots) on the surface of the cell

A498 cells were treated with CsA (10 μM)/TGF-β (5 ng/ml) in separate set of experiments and morphological changes were observed at 48 h. Light microscopy images showed disruption of cell to cell junctions and distinct change to spindle shaped morphology in the cells post CsA/TGF-β treatment (Fig. 1a, b). The Additional file 1: Fig. S1 show that cells progressively acquire the spindle shaped morphology on CsA treatment. Acridine orange staining post 48 h of CsA treatment in A498 cells showed fibroblast like cell shape when observed under fluorescence microscope (Fig. 1a). A significant increase in the average cell size was observed after exposing the cells to CsA as calculated by NIS elements software of the Nikon microscope (Fig. 1c). A significant increase in the average cell size was again observed after exposing the cells to TGF-β as calculated by ImageJ software. (Figure 1d).

To further confirm the biophysical changes in cellular characteristics after EMT induction, we employed atomic force microscopy (AFM) in both control and treated cells. The topography and modulus images obtained are shown in Fig. 1e for the control as well as the treated samples. Topography images clearly indicate that the treated cells become elongated as compared to the control ones. The increase in size of the treated cells in the longitudinal direction was about four times as compared to the control cells. DMT modulus values were extracted from the shaded regions in the modulus images. Corresponding regions are shown as shaded regions in the topography images. Average moduli of the regions were calculated for both the samples. It was found that the modulus of treated cells is less than 25% of that of the control cells.

### Increase in expression of mesenchymal genes in EMT induced cells

**Fig. 2:**
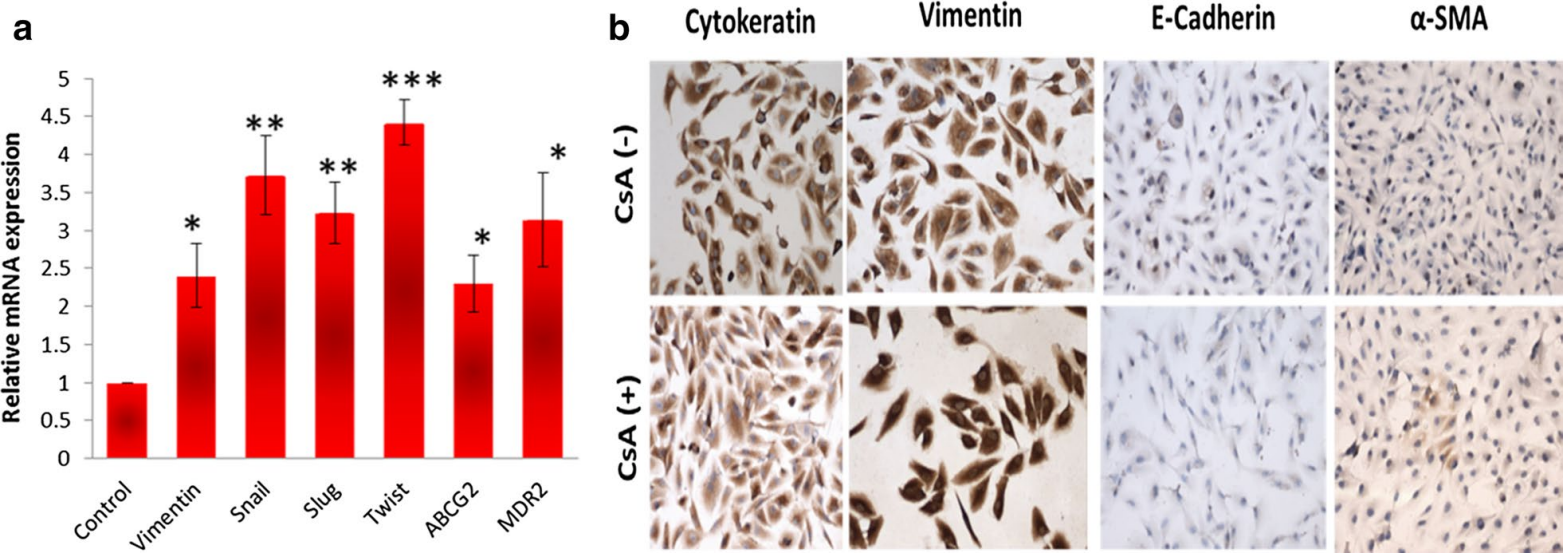
Expression of mesenchymal phenotype and chemoresistance genes in A498 cell line. **a** Cells were treated with CsA and analysed for the expression of various genes (vimentin, snail, slug, twist, ABCG2 and MDR2) evaluated with real time quantitative PCR. **b** Light microscopic images show decreased expression of both pan-cytokeratin and E-cadherin and increased expression of vimentin in cells treated with CsA (10 µM). The cells without treatment were taken as control cells. Magnification—×20

**Fig. 3:**
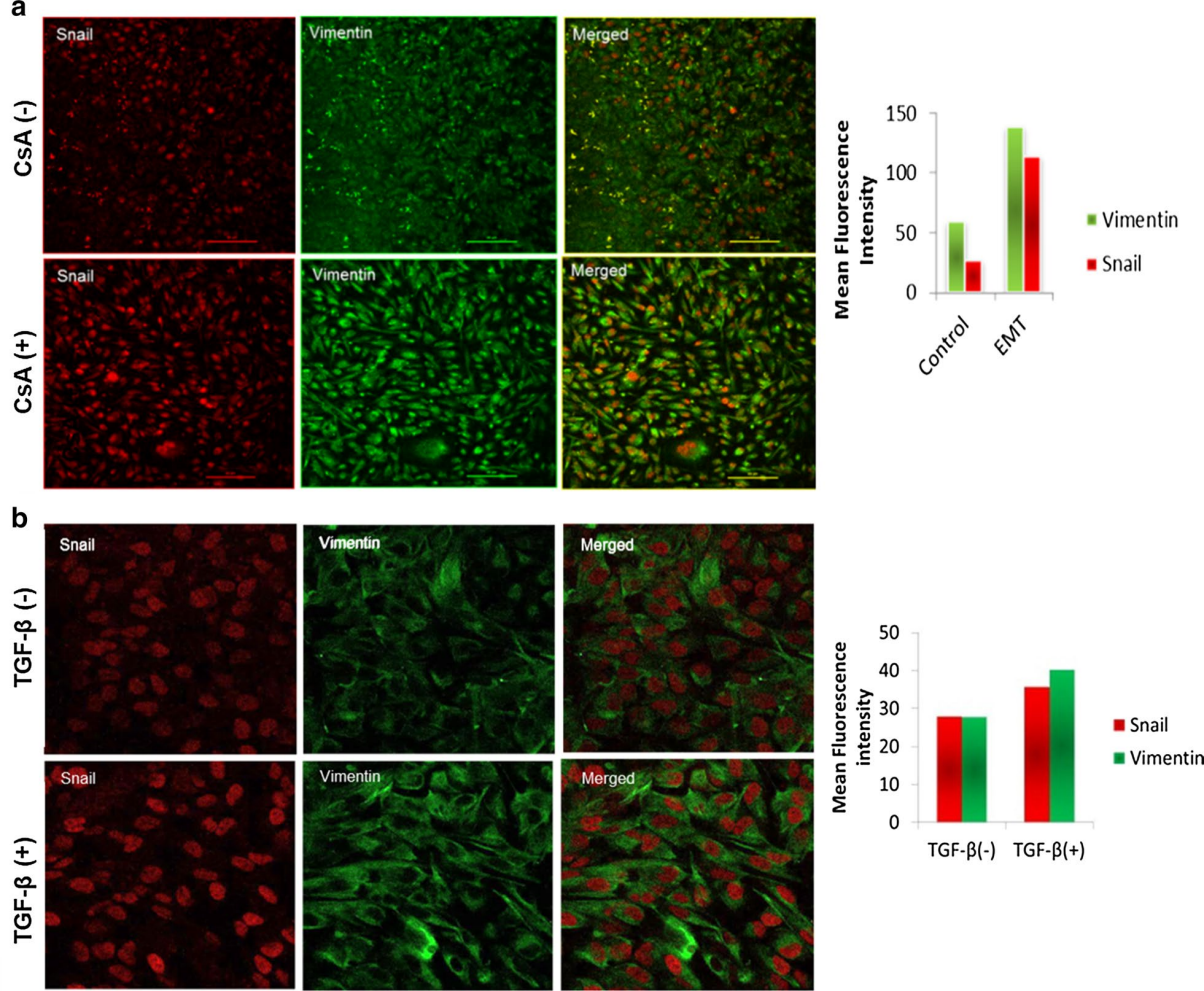
EMT induced cells show high expression of mesenchymal markers: **a** confocal microscopic images show increase in expression of vimentin and snail in CsA (10 µM) treated cells. The cells without treatment were taken as control cells. Magnification—×20, scale bar—60 µm. **b** EMT induced with TGF-β show high expression of mesenchymal markers in A498 cells. Confocal microscopic images show the expression of vimentin and snail. There was an increase in the expression of snail (red) and vimentin (green) in cells treated with TGF-β (5 ng/ml). Magnification—×20. The mean fluorescence intensities in control and treated cells are represented graphically

CsA treated A498 cells showed significantly higher expression of EMT regulators like snail, slug, twist as well as vimentin (2.5- to fourfold) (Fig. 2a). We observed that the ABC transporter, ABCG2 and multidrug resistance gene, MDR1 were also upregulated to a significant level (Fig. 2a). Immunocytochemistry results showed that expression of epithelial markers like E-cadherin and pan-cytokeratin decreased in cells treated with CsA with concomitant rise in the expression of vimentin and α-SMA (Fig. 2b). The EMT signature was also confirmed through immunofluorescence analysis where a higher expression of vimentin and snail were observed in both TGF-β and CsA treated cells (Fig. 3a, b).

### Metastatic potential of EMT induced cell A498 cells

**Fig. 4:**
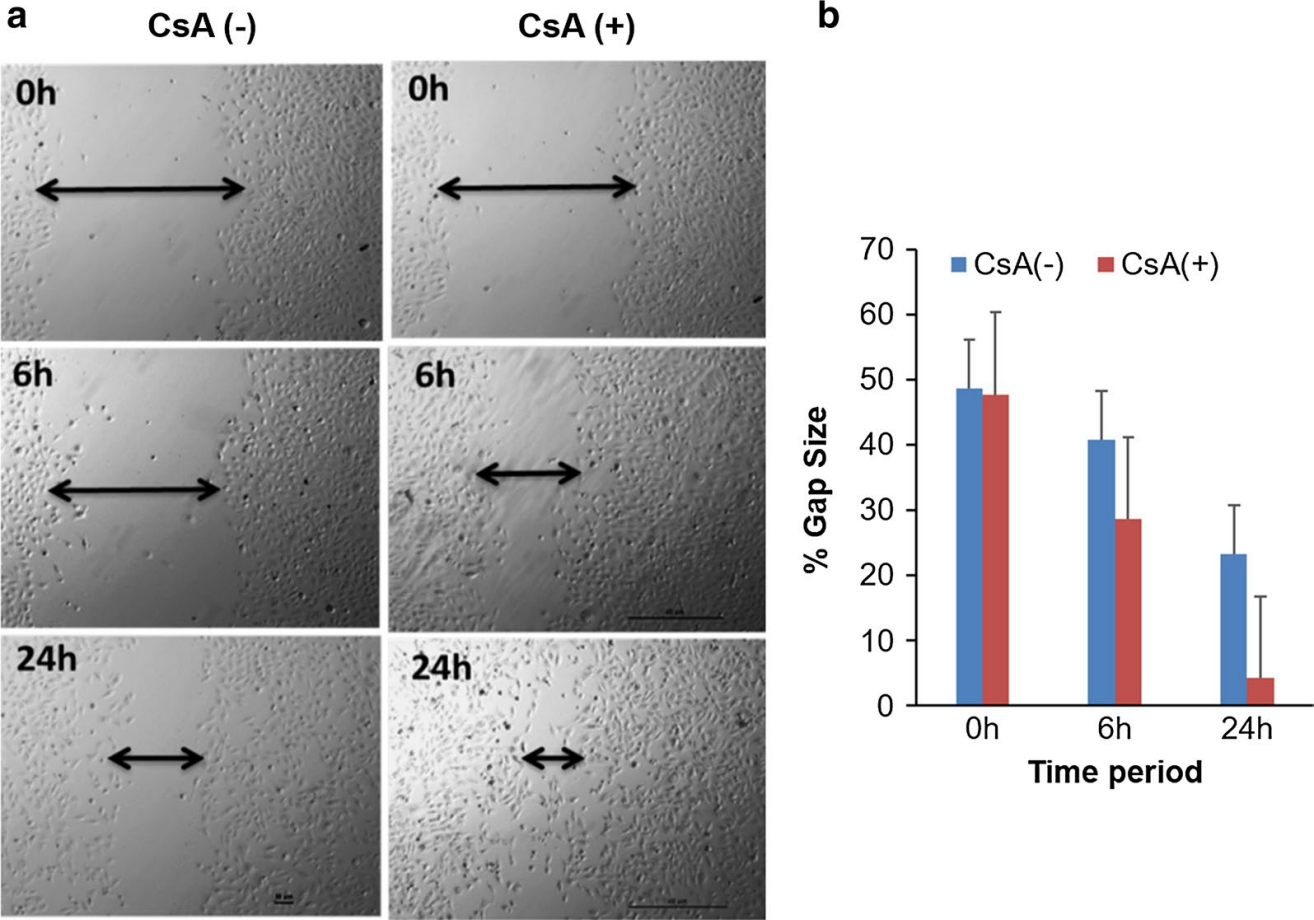
EMT induced cells are more migratory. **a** The migration ability of CsA treated A498 cells and control untreated cells were measured by wound healing assay after 6 and 24 h of wound induction in a 12 well plate. Photos were taken at 0, 6 and 24 h. Magnification—×4. **b** The healing rate was quantified by measurement of the gap size with the T-scratch assay software (open software at http://www.cse-lab.ethz.ch/)

**Fig. 5:**
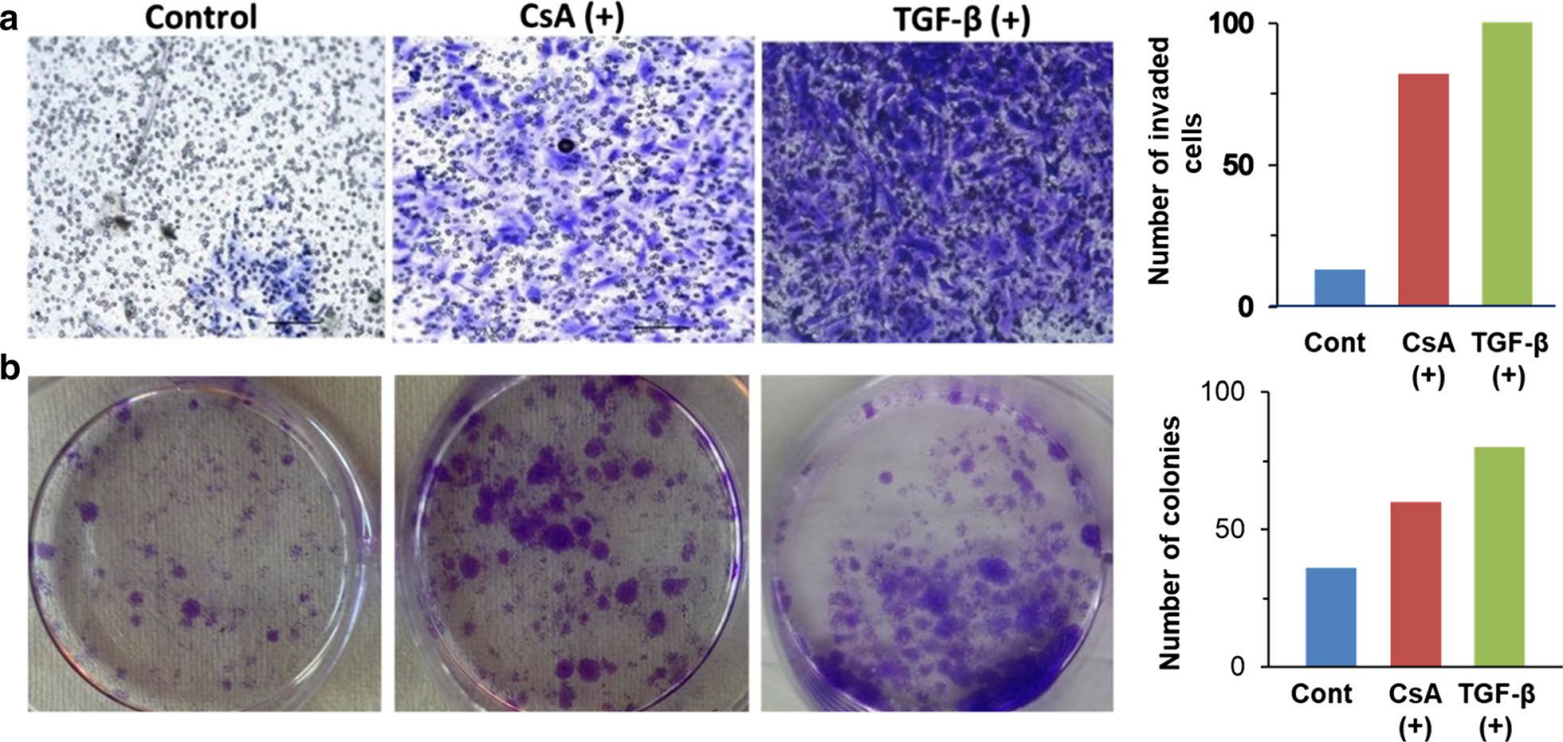
EMT induced cells are more invasive and have high colony forming ability. **a** Transwell invasion assay. 1 × 10^5^ cells were seeded on Matrigel coated inserts. Cells invaded to lower chamber in the absence or presence of CsA or TGF-β were fixed, stained and photographed under bright field microscope (Leica). Magnification—×20. The data is represented graphical alongside. **b** EMT induced cells show higher colony forming ability. Both CsA treated and TGF-β treated cells formed more colonies in comparison to untreated cells. The average number of colonies are shown graphically

Metastasis is characterized by increased invasiveness and migratory ability, so we further analysed whether the CsA and TGF-β treated cells acquired invasive behaviour associated with metastatic cells. Maximum cell migration was observed after 24 h treatment at 10 µM concentration of CsA. Quantification of the open wound area showed that cells treated with CsA migrated faster from both edges of the scratch and filled the gap area in lesser time compared to the control cells (Fig. 4a, b). The Matrigel invasion assay showed that CsA and TGF-β treatment significantly increased the ability of cells to degrade the Matrigel and invade to the opposite side. Crystal violet staining on the lower surface of the *polyethylene terephthalate (PET)* membrane showed higher number of invaded cells following CsA and TGF-β treatment (Fig. 5a). Both CsA and TGF-β treated cells showed higher proliferative capacity as confirmed by the colony formation assay (Fig. 5b).

### Stem cell like properties in EMT induced cells

**Fig. 6:**
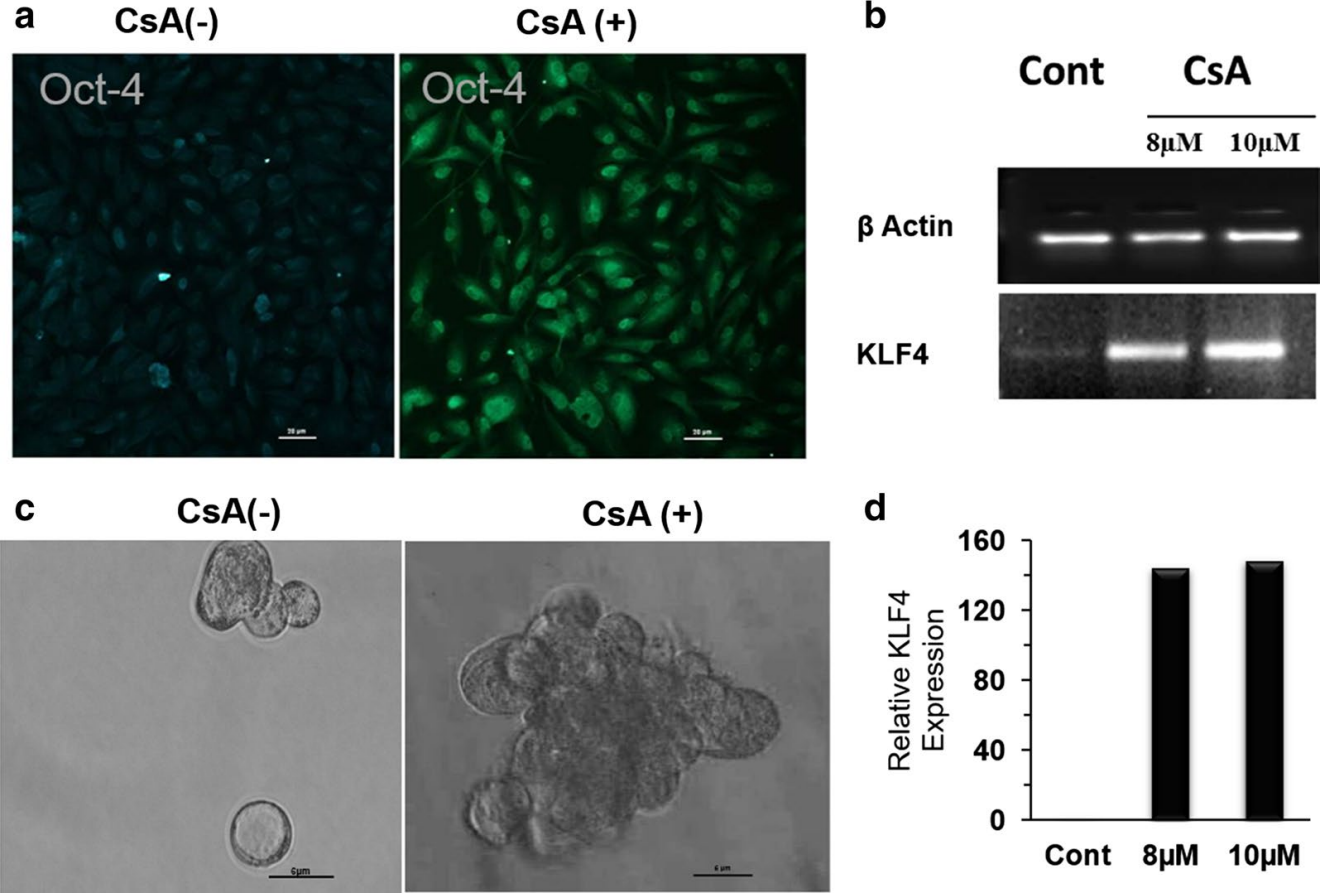
EMT induced cells acquire stem cell-like phenotype: **a** immunofluorescence show expression of the marker for pluripotency i.e. Oct-4 in CsA (10 µM) treated A498 cells. Magnification ×10. Scale bar—20 µm. **b** Agarose gel electrophoresis (2%) showing expression of the stemness gene KLF4 in A498 cells with or without the treatment of CsA (8, 10 μM) and β-actin was used as housekeeping gene. **c** Tumorisphere formation assay: Bright field image showing tumorisphere formation by CsA treated cells grown on non-adherent surface in serum free media. Magnification ×10. **d** Graph showing the KLF4 expression in control and CsA treated A498 cells (8, 10 μM) done by densitometry

**Fig. 7:**
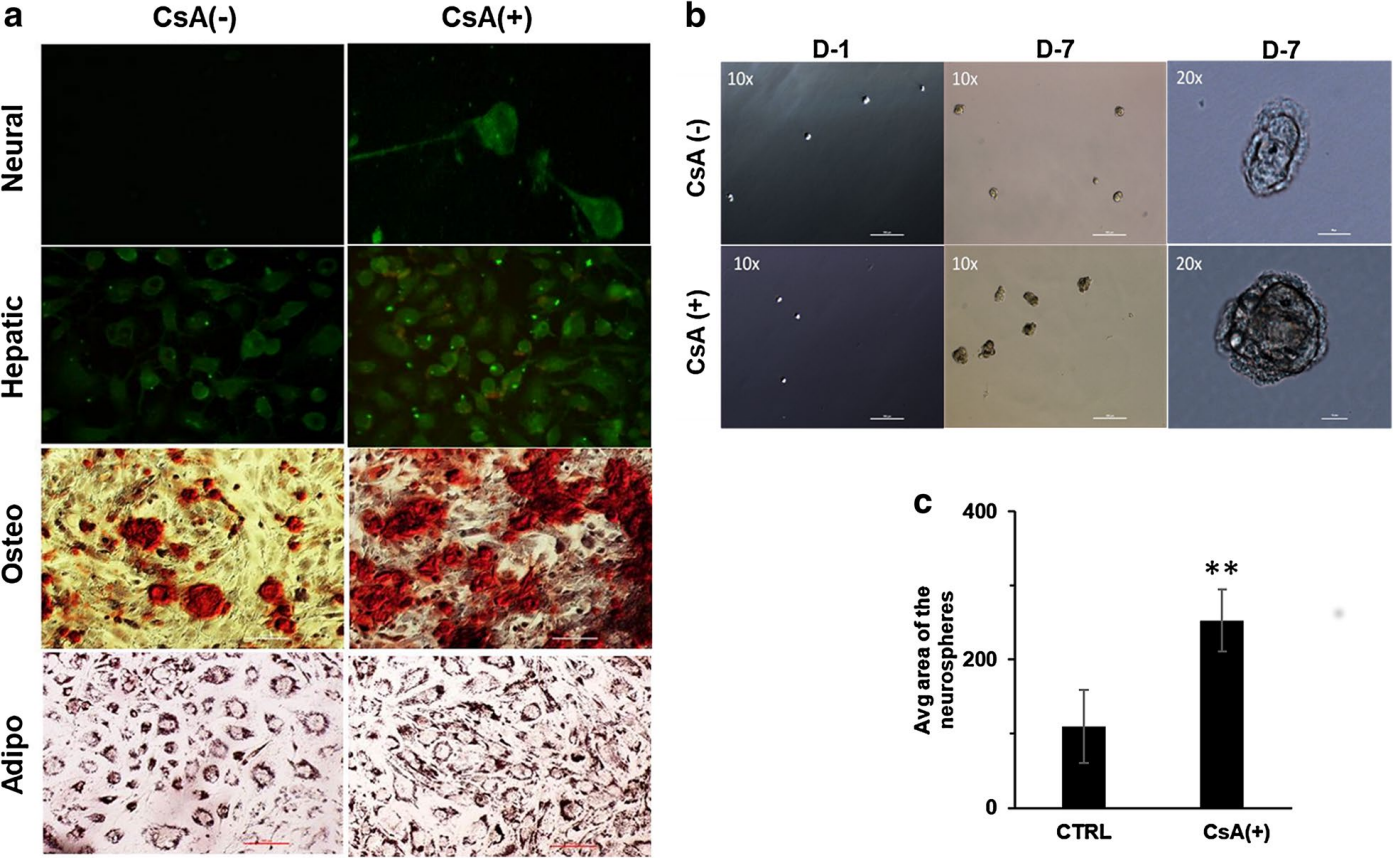
EMT induced cells differentiate into different lineages **a** Neural differentiation on day 21 of CsA-treated (CsA+) and control cells (CsA−). Immunostaining was done with neurofilament as primary antibody (1:50). Magnification 20X. Second panel shows hepatic differentiation on day 28 which was confirmed by LDL uptake. Green color (LDL-Dylight 488) is showing the distribution pattern for LDL receptor on cells while LDL uptake by these cells is shown by red color (LDL-Dylight 549). Magnification 20X. Third panel shows osteogenic differentiation with alizarin red staining of mineralized granules on day 21 of differentiation. Fourth panel shows adipogenic differentiation with oil red O stained lipid droplets in cells, 21 days post-differentiation. Magnification ×10. Scale bar—100 µm). **b** Neurosphere assay in both control (CsA−) and CsA-treated (CsA+) cells at day 1 and day 7. Larger neurospheres can be observed in CsA-treated cells as compared to control cells on day 7. Scale bar—100 µm. Magnified image of day 7 neurospheres are shown on extreme right. Magnification ×20. Scale bar—10 µm. **c** Bar diagram showing comparative analysis of neurosphere area between control (CsA−) and CsA treated (CsA+) cells. **P < 0.001

We checked the expression of pluripotency markers Oct-4 and KLF4 in the EMT induced cells and found significant increase in their expression (Fig. 6a, b, d). EMT undergoing cells also showed increased tendency to form tumor-like spheres on non-adherent surface as compared to control cells (Fig. 6c). Multilineage differentiation potential is a unique feature of pluripotent cells that we confirmed by inducing osteogenic, adipogenic, neural and hepatic differentiation under appropriate stimuli. Neurofilaments which are the characteristic feature of the neuronal cells were found to be expressed in EMT induced cells exposed to neural differentiation media while its expression was almost negligible in bulk A498 cells. Hepatogenic differentiation ability was analysed in cells cultured in hepatogenic differentiation media for 28 days. Accumulation of low density lipo-proteins (LDL) indicated the characteristic feature of hepatocytes. LDL uptake assay using fluorescent labelled antibodies showed higher expression of LDL receptor on EMT induced cells after 28 days. Osteogenic differentiation was confirmed by Alizarin red staining of calcium granules which was higher in EMT induced cells compared to bulk tumor cell population. Adipogenic differentiation was observed with oil red o stain and no significant change in deposition of oil droplets was observed between EMT induced cells and control cells (Fig. 7a). We also observed an increase in density and average size of neurospheres on day 7 in the plate containing EMT induced cells (Fig. 7b, c).

## Discussion

Epithelial–mesenchymal transition (EMT) is an important process during initiation and progression of the metastatic cascade. EMT pathway can have therapeutic implications in cancer treatment. The tendency to metastasize and resistance to chemotherapy is the main cause of low survival in most of the malignancies including RCC. It was recently hypothesized that drug resistance, disease progression, and recurrence are mediated by stem cell-like cancer cells also referred to as cancer stem cells/tumor-initiating cells (CSCs/TICs) [13, 14]. The phenotypic plasticity displayed by CSCs may arise in the process of and/or undergo EMT. Isolating the CSC population in each tumor type is a major challenge due to its small subpopulation. CD 105 was identified as a biomarker for renal CSCs [15] and another study suggested that it can also serve as a functional target for therapeutic intervention [16]. Subsequent gene profiling and pathway analysis of CD105 high cells showed high activation of TGF-beta in these cells [17]. TGF beta is an inducer of EMT and promotes tumorigenesis and metastasis by EMT [18–20]. Previously, it was demonstrated that exposure to a clinically relevant dose of the immunosuppressant agent CsA, led to an EMT in human proximal tubular cells through TGF-β [21]. In this context, we pharmacologically induced EMT in RCC cell line A498 with CsA and TGF-β. We observed that the biological behaviour of TGF-β and CsA treated cells were consistent with that of mesenchymal cells, including a change in morphological features, lower adhesion and higher in vitro migratory capacity. The treatment caused higher expression of EMT regulators like snail, slug, twist as well as vimentin along with deceased expression of E cadherin. These modulations in cellular phenotype cause increased motility, invasiveness and resistance to apoptosis of cancer cells [22]. There is loss of E cadherin protein during EMT to occur and promote metastasis [23]. The loss of E-cadherin is an integral step during the EMT process and a key feature of metastatic cells. Without the tight adherent junctions which bind the tissues together, individual cells are free to migrate, a crucial requirement for cancer metastasis. The shift from cytokeratin intermediate filaments to vimentin and expression of other mesenchymal markers like snail, slug and twist suggested the occurrence of an EMT-like event in cells treated with TGF-β/CsA. The morphology of the cancer progenitor cells changes during the process of EMT. Rearrangement of the cytoskeleton is an essential feature during EMT. Expression of vimentin, which is an important constituent of the cytoskeleton, goes up in many types of cancer during EMT and in the stromal cells of several cancers [24].

The adaptation for invasiveness and metastasis in cancer cells involve mechanical softening and modification in interactions with ECM. These modifications enable the cancer cells to migrate from their primary site to invade another secondary site. Young’s modulus is an excellent representative of cell’s elasticity/stiffness and evaluation of this modulus by atomic force microscopy offers an excellent approach to correlate the malignancy and deformity of cancer cells [25, 26]. A decreased Young’s modulus (< 25%) in case of EMT induced cells as compared to control cells clearly reflects a softening in EMT induced cells, an adaptation for invasion and metastasis.

It has been known that there is an intricate relationship between cell shape and function. So, a change in the cell after induction of EMT can be extrapolated to changes in cellular physiology due to interplay of genes and proteins. Cells that are characterized as invasive and metastatic are typically able to invade matrigel matrix which mimics the cell basement membrane [27]. The increased migration and stronger ability to invade through matrigel further indicated that these cells of epithelial origin have acquired metastatic phenotype. The cells treated with TGF-β and CsA also showed higher colony formation signifying an increased proliferative potential compared to bulk A498 cells. We have identified several changes in both protein and mRNA expression indicative of EMT in TGF-β and CsA treated cells. The tumorispheres formation by A498 cells post CsA treatment, further confirmed that these cells have attained an EMT-like phenotype. The formation of tumorispheres in non-adherent culture is a useful functional approach to enrich the potential subpopulation of CSC. The sphere forming cells are reported to show higher proliferation and self-renewal ability [28]. Self-renewal of undifferentiated stem cells is mainly regulated by octamer binding transcription factor 4 (Oct-4) protein [29]. Thus, our observation of an increase in the oct4 expression in CsA treated cell further highlights the fact that these cells acquire a stem cell-like phenotype. A study by Takahashi and Yamanaka reported that kruppel-like factor 4 (KLF4) is indispensable for maintenance of stem cells [30]. Fang et al. also elucidated that consistent overexpression of KLF4 led to increase in population of the cancer stem cells [31]. mRNA expression of KLF4 was found to be upregulated in TGF-β/CsA treated cells. Sphere formation ability and increase in the expression of Oct-4 in CsA treated cells may account for an increase in stemness and differentiation potential of these cells.

The ability for osteogenic, hepatogenic and neural differentiation depicts the multilineage potential of EMT cells. There was significant rise in expression of each of osteogenic, hepatogenic and neural phenotype in CsA treated cells compared to control during lineage specific induction for differentiation. The ability for multilineage differentiation by EMT induced cells implies the acquisition of stem cell-like characteristics by these cells. The expression of oil red O in the control cells may be due to the fact that the clear cell RCC is reported to contain lipid droplets and histologically resemble adipocytes [32].

EMT phenomenon is reported to be associated with drug resistance in many tumors. In our study, we observed higher expression of ATP-binding cassette sub-family G member 2 (ABCG2) and multidrug resistance protein 1 (MDR1) genes in CsA treated cells. In accordance to our study, Saxena et al. demonstrated that EMT transcription factors cause multidrug resistance by upregulation of ABC transporters. Furthermore, these authors reported that ABC transporters harbour many binding sites for EMT inducing transcription factors, like Snail, and FOXC2, Twist etc. which ultimately induce EMT by enhancing the activity at promoter site of ABC transporters [33].

**Fig. 8:**
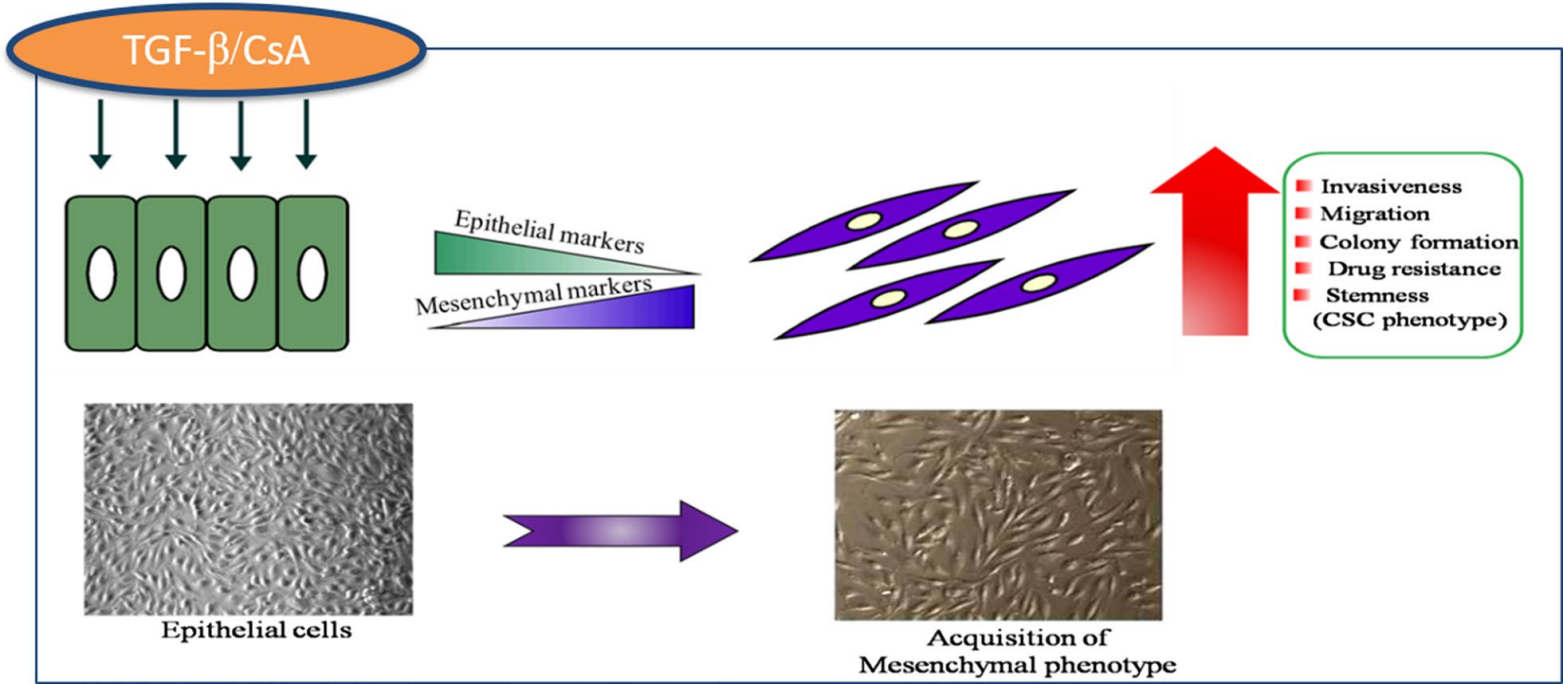
Schematic representation of EMT in renal carcinoma cells: A498 cells treated with CsA/TGF-β show downregulation of epithelial markers and upregulation of mesenchymal markers. This is followed by concomitant increase in invasiveness, migration, drug resistance and expression of stem cell like phenotype. This suggests that transition from epithelial to mesenchymal phenotype leads to acquisition of cancer stem cell phenotype

The machinery of EMT seems to be connected directly to formation and maintenance of CSCs leading to tumor metastases, drug resistance and recurrence [34]. Our results suggest that a mesenchymal-like phenotype following CsA/TGF-β treatment is due to EMT. Walsh et al. have demonstrated that administration of CsA to animal model of cutaneous squamous cell carcinoma alters the phenotype to an invasive and aggressive tumor-type by enhancing EMT through the TGFβ1 signaling pathway [35]. These cells behave as de-differentiated stem cells isolated from normal or neoplastic cells. Previously, Mani et al. induced EMT in human, non-tumorigenic mammary epithelial cells using TWIST or SNAIL transcription factors. They observed that EMT in breast cancer cells generated a subpopulation of cells with properties of stem cells which may be responsible for drug resistance and recurrence [36]. This small subpopulation of cells within the tumor bulk was categorised as the cancer stem cells. These may serve as an in vitro platform for improving the present clinical therapy of cancer since cells become invasive and resistant to apoptosis after acquiring mesenchymal phenotype. We show that the pharmacologically induced EMT cells of A498 behave as cancer stem-like cells **(**Fig. 8). In some cases, CSCs have been identified as independent predictor of progression-free survival and overall survival [37, 38]. These cells may therefore serve as an in vitro platform for improving the present clinical therapy of cancer since cells become invasive and resistant to apoptosis after acquiring mesenchymal phenotype. As observed in our study, many clinical studies also detected the presence of EMT molecular markers in the CSCs. Breast cancer patients derived CSCs showed decreased expression of epithelial markers and increased mesenchymal markers [39–42]. In another study, Twist1 expression in breast cancer patients correlated with early distant relapse [43]. CSCs isolated from hepatocellular carcinoma (HCC) patients with metastasis showed higher expression of Snail1 transcripts compared to patients with no metastasis [44]. One caveat of the studies involving CSCs is that, the CSCs constitute a very minor fraction of the total cancer cells and hence may be missed out during isolation in vivo [45–47]. Thus, there is a need to explore the various methods to generate a CSC-like population in vitro and study the potential utility of CSCs in targeting the metastatic spread and cancer recurrence.

Our results suggest that CsA and TGF-β induced EMT like changes may help to study the mechanisms regulating drug resistance and invasiveness. Data on patients under CsA treatment have suggested that long-term CsA may lead to an increased susceptibility to certain tumours and tumor metastasis [48]. Present study endorses the view of risk of malignancy in transplant patients and provides evidence that cyclosporine can enhance cancer in renal transplant patients and can make it more aggressive. Rate of complete response in patients with metastatic RCC treated with targeted agents is very low and, better understanding of the pathogenic mechanisms of RCC brings hope for new strategies. Thus, targeting the pathways that promote EMT may lead to novel therapeutic strategies for the prevention and treatment of cancer, especially following organ transplantation.

## Conclusion

Our study delineates the precise role of EMT in RCC carcinogenesis and metastasis. The expression profile and other biological properties suggest that EMT can be pharmacologically induced in vitro by CsA or TGF-beta and these EMT-induced cells acquire CSC-like phenotype. The results of our study support the rationale for future EMT-directed therapeutic approaches for RCC patients.

## Additional file

**Additional file 1: Fig. S1.** Time dependent change in cell morphology with CsA: the A498 cells were treated with CsA for 6, 12 and 24 h and the morphological change was compared with corresponding untreated control to assess EMT. The result was confirmed by the expression of various epithelial and mesenchymal markers at the selected time and dose.

## Abbreviations

EMT: epithelial to mesenchymal transition
CsA: cyclosporine A
TGF-β: transforming growth factor beta
CSC: cancer stem cell
hEGF: human epidermal growth factor
bFGF: basic fibroblast growth factor
NFM: neurofilament
Oct-4: Octamer binding transcription factor-4
ABCG2: ATP binding cassette sub-family G member 2
MDR1: multidrug resistance protein 1

## References

[1] Nieto MA, Huang RY, Jackson RA, Thiery JP (2016). Emt: 2016. Cell.

[2] Li QS, Lee GY, Ong CN, Lim CT (2008). AFM indentation study of breast cancer cells. Biochem Biophys Res Commun.

[3] Hojo M, Morimoto T, Maluccio M, Asano T, Morimoto K, Lagman M, Shimbo T, Suthanthiran M (1999). Cyclosporine induces cancer progression by a cell-autonomous mechanism. Nature.

[4] Durnian JM, Stewart RM, Tatham R, Batterbury M, Kaye SB (2007). Cyclosporin-A associated malignancy. Clin Ophthalmol.

[5] Shima T, Yamamoto Y, Okuhira H, Mikita N, Furukawa F (2016). A patient with refractory psoriasis who developed sebaceous carcinoma on the neck during cyclosporine therapy and showed rapid progression. Case Rep Dermatol.

[6] Wu X, Nguyen BC, Dziunycz P, Chang S, Brooks Y, Lefort K, Hofbauer GF, Dotto GP (2010). Opposing roles for calcineurin and ATF3 in squamous skin cancer. Nature.

[7] Cotrim P, Martelli-Junior H, Graner E, Sauk JJ, Coletta RD (2003). Cyclosporin A induces proliferation in human gingival fibroblasts via induction of transforming growth factor-beta1. J Periodontol.

[8] Moustakas A, Heldin CH (2012). Induction of epithelial–mesenchymal transition by transforming growth factor beta. Semin Cancer Biol.

[9] Malfettone A, Soukupova J, Bertran E, Crosas-Molist E, Lastra R, Fernando J, Koudelkova P, Rani B, Fabra A, Serrano T, Ramos E, Mikulits W, Giannelli G, Fabregat I (2017). Transforming growth factor-beta-induced plasticity causes a migratory stemness phenotype in hepatocellular carcinoma. Cancer Lett.

[10] Massague J (2012). TGFbeta signalling in context. Nat Rev Mol Cell Biol.

[11] Geback T, Schulz MM, Koumoutsakos P, Detmar M (2009). TScratch: a novel and simple software tool for automated analysis of monolayer wound healing assays. Biotechniques.

[12] Zhang Y, Yan W, Jung YS, Chen X (2012). Mammary epithelial cell polarity is regulated differentially by p73 isoforms via epithelial-to-mesenchymal transition. J Biol Chem.

[13] Adorno-Cruz V, Kibria G, Liu X, Doherty M, Junk DJ, Guan D, Hubert C, Venere M, Mulkearns-Hubert E, Sinyuk M, Alvarado A, Caplan AI, Rich J, Gerson SL, Lathia J, Liu H (2015). Cancer stem cells: targeting the roots of cancer, seeds of metastasis, and sources of therapy resistance. Cancer Res.

[14] Czarnecka M, Cezary S (2013). Renal cell carcinoma cancer stem cells as therapeutic targets. Curr Signal Transduct Ther.

[15] Bussolati B, Bruno S, Grange C, Ferrando U, Camussi G (2008). Identification of a tumor-initiating stem cell population in human renal carcinomas. FASEB J.

[16] Hu J, Guan W, Liu P, Dai J, Tang K, Xiao H, Qian Y, Sharrow AC, Ye Z, Wu L, Xu H (2017). Endoglin is essential for the maintenance of self-renewal and chemoresistance in renal cancer stem cells. Stem Cell Rep.

[17] Tang FR, Wang JW, Tang Z, Kang M, Deng QL, Yu JM (2016). Quality of life and its association with physical activity among different types of cancer survivors. PLoS ONE.

[18] Thiery JP, Acloque H, Huang RY, Nieto MA (2009). Epithelial–mesenchymal transitions in development and disease. Cell.

[19] Heldin CH, Vanlandewijck M, Moustakas A (2012). Regulation of EMT by TGFbeta in cancer. FEBS Lett.

[20] Thiery JP (2002). Epithelial–mesenchymal transitions in tumour progression. Nat Rev Cancer.

[21] Slattery C, Campbell E, McMorrow T, Ryan MP (2005). Cyclosporine A-induced renal fibrosis—a role for epithelial–mesenchymal transition. Am J Pathol.

[22] Li L, Li W (2015). Epithelial-mesenchymal transition in human cancer: comprehensive reprogramming of metabolism, epigenetics, and differentiation. Pharmacol Ther.

[23] Onder TT, Gupta PB, Mani SA, Yang J, Lander ES, Weinberg RA (2008). Loss of E-cadherin promotes metastasis via multiple downstream transcriptional pathways. Cancer Res.

[24] Abraham E, Marincola FM, Chen Z, Wang X (2012). Clinical and translational medicine: integrative and practical science. Clin Transl Med.

[25] Guck J, Schinkinger S, Lincoln B, Wottawah F, Ebert S, Romeyke M, Lenz D, Erickson HM, Ananthakrishnan R, Mitchell D, Kas J, Ulvick S, Bilby C (2005). Optical deformability as an inherent cell marker for testing malignant transformation and metastatic competence. Biophys J.

[26] Xu W, Mezencev R, Kim B, Wang L, McDonald J, Sulchek T (2012). Cell stiffness is a biomarker of the metastatic potential of ovarian cancer cells. PLoS ONE.

[27] Albini A, Iwamoto Y, Kleinman HK, Martin GR, Aaronson SA, Kozlowski JM, McEwan RN (1987). A rapid in vitro assay for quantitating the invasive potential of tumor cells. Can Res.

[28] Cao L, Zhou Y, Zhai B, Liao J, Xu W, Zhang R, Li J, Zhang Y, Chen L, Qian H, Wu M, Yin Z (2011). Sphere-forming cell subpopulations with cancer stem cell properties in human hepatoma cell lines. BMC Gastroenterol.

[29] Zhang ZN, Chung SK, Xu Z, Xu Y (2014). Oct4 maintains the pluripotency of human embryonic stem cells by inactivating p53 through Sirt1-mediated deacetylation. Stem Cells.

[30] Takahashi K, Yamanaka S (2006). Induction of pluripotent stem cells from mouse embryonic and adult fibroblast cultures by defined factors. Cell.

[31] Yu F, Li J, Chen H, Fu J, Ray S, Huang S, Zheng H, Ai W (2011). Kruppel-like factor 4 (KLF4) is required for maintenance of breast cancer stem cells and for cell migration and invasion. Oncogene.

[32] Rezende RB, Drachenberg CB, Kumar D, Blanchaert R, Ord RA, Ioffe OB, Papadimitriou JC (1999). Differential diagnosis between monomorphic clear cell adenocarcinoma of salivary glands and renal (clear) cell carcinoma. Am J Surg Pathol.

[33] Saxena M, Stephens MA, Pathak H, Rangarajan A (2011). Transcription factors that mediate epithelial–mesenchymal transition lead to multidrug resistance by upregulating ABC transporters. Cell Death Dis.

[34] Zhang L, Jiao M, Wu K, Li L, Zhu G, Wang X, He D, Wu D (2014). TNF-alpha induced epithelial mesenchymal transition increases stemness properties in renal cell carcinoma cells. Int J Clin Exp Med.

[35] Walsh SB, Xu J, Xu H, Kurundkar AR, Maheshwari A, Grizzle WE, Timares L, Huang CC, Kopelovich L, Elmets CA, Athar M (2011). Cyclosporine a mediates pathogenesis of aggressive cutaneous squamous cell carcinoma by augmenting epithelial–mesenchymal transition: role of TGFbeta signaling pathway. Mol Carcinog.

[36] Mani SA, Guo W, Liao MJ, Eaton EN, Ayyanan A, Zhou AY, Brooks M, Reinhard F, Zhang CC, Shipitsin M, Campbell LL, Polyak K, Brisken C, Yang J, Weinberg RA (2008). The epithelial–mesenchymal transition generates cells with properties of stem cells. Cell.

[37] Cohen SJ, Punt CJ, Iannotti N, Saidman BH, Sabbath KD, Gabrail NY, Picus J, Morse M, Mitchell E, Miller MC, Doyle GV, Tissing H, Terstappen LW, Meropol NJ (2008). Relationship of circulating tumor cells to tumor response, progression-free survival, and overall survival in patients with metastatic colorectal cancer. J Clin Oncol.

[38] Christoffersen NR, Silahtaroglu A, Orom UA, Kauppinen S, Lund AH (2007). miR-200b mediates post-transcriptional repression of ZFHX1B. RNA.

[39] Mego M, Mani SA, Lee BN, Li C, Evans KW, Cohen EN, Gao H, Jackson SA, Giordano A, Hortobagyi GN, Cristofanilli M, Lucci A, Reuben JM (2012). Expression of epithelial–mesenchymal transition-inducing transcription factors in primary breast cancer: the effect of neoadjuvant therapy. Int J Cancer.

[40] Raimondi C, Gradilone A, Naso G, Vincenzi B, Petracca A, Nicolazzo C, Palazzo A, Saltarelli R, Spremberg F, Cortesi E, Gazzaniga P (2011). Epithelial–mesenchymal transition and stemness features in circulating tumor cells from breast cancer patients. Breast Cancer Res Treat.

[41] Kallergi G, Papadaki MA, Politaki E, Mavroudis D, Georgoulias V, Agelaki S (2011). Epithelial to mesenchymal transition markers expressed in circulating tumour cells of early and metastatic breast cancer patients. Breast Cancer Res.

[42] Aktas B, Tewes M, Fehm T, Hauch S, Kimmig R, Kasimir-Bauer S (2009). Stem cell and epithelial–mesenchymal transition markers are frequently overexpressed in circulating tumor cells of metastatic breast cancer patients. Breast Cancer Res.

[43] Watson MA, Ylagan LR, Trinkaus KM, Gillanders WE, Naughton MJ, Weilbaecher KN, Fleming TP, Aft RL (2007). Isolation and molecular profiling of bone marrow micrometastases identifies TWIST1 as a marker of early tumor relapse in breast cancer patients. Clin Cancer Res.

[44] Min AL, Choi JY, Woo HY, Kim JD, Kwon JH, Bae SH, Yoon SK, Shin SH, Chung YJ, Jung CK (2009). High expression of Snail mRNA in blood from hepatocellular carcinoma patients with extra-hepatic metastasis. Clin Exp Metastasis.

[45] Kang Y, Pantel K (2013). Tumor cell dissemination: emerging biological insights from animal models and cancer patients. Cancer Cell.

[46] Pantel K, Alix-Panabieres C (2010). Circulating tumour cells in cancer patients: challenges and perspectives. Trends Mol Med.

[47] Paterlini-Brechot P, Benali NL (2007). Circulating tumor cells (CTC) detection: clinical impact and future directions. Cancer Lett.

[48] Haberal M, Karakayali H, Emiroglu R, Basaran O, Moray G, Bilgin N (2002). Malignant tumors after renal transplantation. Artif Organs.

