# Supplementary figures and images for: Epithelial to mesenchymal transition induces stem cell like phenotype in renal cell carcinoma cells
Source: Cancer Cell Int. 2018 Apr 11;18:57. doi: 10.1186/s12935-018-0555-6 (PMC5896088; doi:10.1186/s12935-018-0555-6)

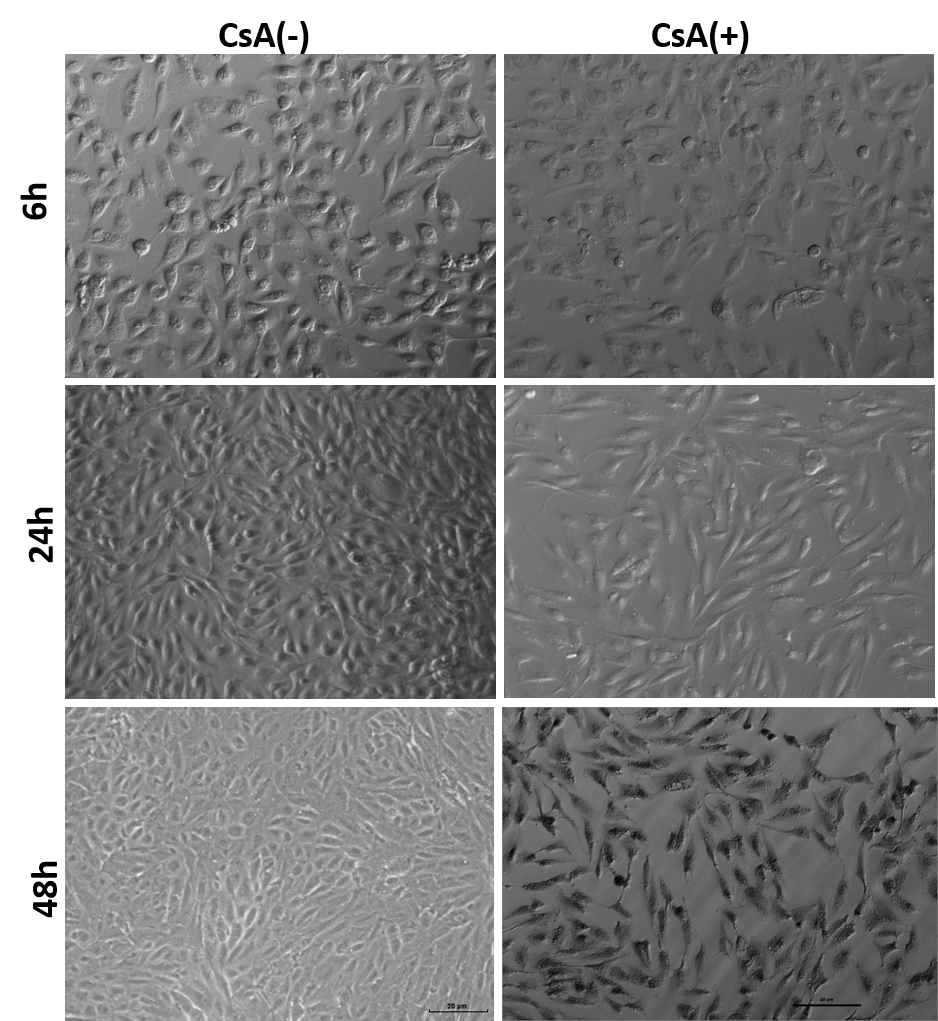

Supplement: Supplementary file 1 — Additional file 1: Fig. S1. Time dependent change in cell morphology with CsA: the A498 cells were treated with CsA for 6, 12 and 24 h and the morphological change was compared with corresponding untreated control to assess EMT. The result was confirmed by the expression of various epithelial and mesenchymal markers at the selected time and dose. [file 12935_2018_555_MOESM1_ESM.tif]
